# Potential causes and consequences of behavioural resilience and resistance in malaria vector populations: a mathematical modelling analysis

**DOI:** 10.1186/1475-2875-13-97

**Published:** 2014-03-14

**Authors:** Gerry F Killeen, Nakul Chitnis

**Affiliations:** 1 Ifakara Health Institute, Environmental Health and Ecological Sciences Thematic Group, PO Box 53, Ifakara, Kilombero, Morogoro, United Republic of Tanzania; 2 Department of Vector Biology, Liverpool School of Tropical Medicine, Liverpool L3 5QA, UK; 3 Department of Epidemiology and Public Health, Swiss Tropical and Public Health Institute, Socinstrasse 57, Basel 4002, Switzerland; 4 University of Basel, Basel, Switzerland; 5 Fogarty International Center, National Institutes of Health, Bethesda, MD 20892, USA

**Keywords:** *Plasmodium*, *Anopheles*, Malaria, Behaviour, Resistance, Vector control, Endophagic, Exophagic, Endophilic, Exophilic

## Abstract

**Background:**

The ability of mosquitoes to evade fatal exposure to insecticidal nets and sprays represents the primary obstacle to eliminating malaria. However, it remains unclear which behaviours are most important for buffering mosquito and parasite populations against vector control.

**Methods:**

Simulated life histories were used to compare the impact of alternative feeding behaviour strategies upon overall lifetime feeding success, and upon temporal distributions of successful feeds and biting rates experienced by unprotected humans, in the presence and absence of insecticidal nets. Strictly nocturnal preferred feeding times were contrasted with 1) a wider preference window extending to dawn and dusk, and 2) crepuscular preferences wherein foraging is suppressed when humans sleep and can use nets but is maximal immediately before and after. Simulations with diversion and mortality parameters typical of endophagic, endophilic African vectors, such as *Anopheles gambiae* and *Anopheles funestus*, were compared with those for endophagic but exophilic species, such as *Anopheles arabiensis*, that also enter houses but leave earlier before lethal exposure to insecticide-treated surfaces occurs.

**Results:**

Insecticidal nets were predicted to redistribute successful feeding events to dawn and dusk where these were included in the profile of innately preferred feeding times. However, predicted distributions of biting unprotected humans were unaffected because extended host-seeking activity was redistributed to innately preferred feeding times. Recently observed alterations of biting activity distributions therefore reflect processes not captured in this model, such as evolutionary selection of heritably modified feeding time preferences or phenotypically plastic expression of feeding time preference caused by associative learning. Surprisingly, endophagy combined with exophily, among mosquitoes that enter houses but then feed and/or rest briefly before rapidly exiting, consistently attenuated predicted insecticide impact more than any feeding time preference trait.

**Conclusions:**

Regardless of underlying cause, recent redistributions of host-biting activity to dawn and dusk necessitate new outdoor control strategies. However, persistently indoor-feeding vectors, that evade intradomiciliary insecticide exposure, are at least equally important. Fortunately, recent evaluations of occupied houses or odour-baited stations, with baffled entrances that retain *An. arabiensis* within insecticide-treated structures, illustrate how endophagic but exophilic vectors may be more effectively tackled using existing insecticides.

## Background

The most potent and important malaria vectors in Africa, namely *Anopheles gambiae* and *Anopheles funestus*, are readily vulnerable to population control, or even elimination, with indoor residual spraying of insecticides (IRS) or long-lasting insecticidal nets (LLINs) because they predominantly feed on humans at times of the night when they are asleep inside houses [1-5]. A number of important primary American and Asian vectors have also been successfully suppressed or eliminated with IRS or LLINs because they are similarly dependent upon feeding on humans while they are asleep indoors at night [1,3-6]. However, atypical or changing behavioural patterns have recently been reported for several vector species in settings where scale up of LLINs, IRS or mosquito-proofed housing has been associated with increased proportions of observed attacks on humans occurring in the evenings or mornings when most people are active outdoors and therefore unprotected [7-13]. Furthermore, the remarkably persistent African species *Anopheles arabiensis* also avoids fatal insecticide exposure through a less obvious form of behavioural evasion, by entering but then rapidly exiting houses containing IRS and LLINs [14-16]. Moreover, this important contributor to residual transmission in Africa [8,17-20] can also safely feed upon insecticide-treated cattle by doing so in less than one minute [21] in a manner that is remarkably reminiscent of the two-minute resting periods on walls and ceilings which were implicated in the failure of IRS to eliminate malaria transmission by *Anopheles darlingi*, *Anopheles punctimacula* and *Anopheles nuneztovari* from the Americas [22].

Many of these observations of unusual or apparently changed behaviour may well be attributed to altered taxonomic composition caused by selective suppression of the most behaviourally vulnerable taxa, and/or by altered expression of pre-existing plastic behavioural phenotypes in response to modified resource availability patterns [3,5,23]. It therefore remains unclear whether these observations represent emerging behavioural resistance in the strict sense, meaning that innate behavioural preferences have been selected for by LLINs/IRS which allow mosquitoes to increasingly evade fatal contact with them [5,24], or merely the modified expression of pre-existing, phenotypically plastic evasion behaviours that might be better described as resilience [3,25]. It also remains unclear which specific behaviours are most important for buffering mosquito populations and malaria parasite transmission against the impact of vector control. Here, these important issues in malaria vector control are explored by applying a process-explicit model of mosquito behaviour and lifetime feeding history to compare the impact of alternative feeding behaviour strategies upon overall feeding success, as well as the temporal distributions of successful feeds across entire communities and biting activity upon unprotected humans, in the presence and absence of LLINs.

## Methods

**Figure 1:**
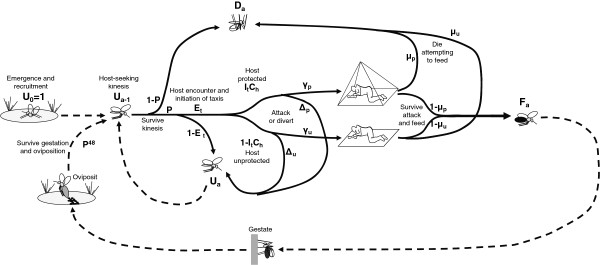
**A schematic representation of the model structure, processes and equations.** Solid arrows represent processes occurring within a single age interval of one hour (a) while dashed arrows represent processes linking one age interval to a later one. See Table 1 for detailed description of the basic input parameter definitions and values, as well as table 2 for the definitions and equations for derived process, state and life history parameters.

All of the simulations described here were accomplished by adapting an existing vector life history model [26] to operate on hourly, rather than daily, time increments (Figure 1). Unless stated otherwise here, all definitions, equations and parameter values (Tables 1 and 2) remain exactly as originally described in detail elsewhere [26] and subsequently applied to a variety of different analyses [3,4,16,27-29].

**Table 1:** Definitions and values for basic input parameters

**Symbol**	**Definition**	**Values**	**Source and justification**
*a*	Age of a mosquito in hours at the end of a given hour-long time interval	1,2,3....720	Defined here
*C*_*h*_	Demographic coverage of humans with long-lasting insecticidal nets, expressed as the proportion of humans that use one each night	0 or 0.8	Complete absence or World Health Organization targets [39]
*d*	Age of a mosquito in days, rounded down to whole days, at the end of a given hour-long time interval	1,2,3....720	Defined here
*Δ*_*u*_	Probability of mosquito diverting away from attacking an encountered unprotected human host	0.1	[26,40]
*Δ*_*p*_	Probability of mosquito diverting away from attacking an encountered human host who is protected by using a long-lasting insecticidal net at that time	0.6 or 0.8	Stereotypical values for normal and behaviourally evasive vectors, respectively [14-16,41]
*E*_*max*_	Rate at which host-seeking mosquitoes encounter human hosts when fully active	0.181 encounters · host seeking mosquito^-1^ · hour^-1^	Derived [31] from field studies of wild *An. arabiensis* in Tanzania [42].
*I*_*t*_	Proportion of human population indoors and asleep where they can be protected by using long-lasting insecticidal nets	0 to 1	Assumed to vary over the course of each 24-hour day to reflect a stereotypical average of survey results from rural Africa [2]
*μ*_*u*_	Probability of mosquito dying as a result of attacking an encountered unprotected human host	0.1	[26,40]
*μ*_*p*_	Probability of mosquito dying as a result of attacking an encountered human host who is protected by using a long-lasting insecticidal net at that time	0.6 or 0.2	Stereotypical values for normal and behaviourally evasive vectors, respectively [15,16,41]
*N*_*h*_	Number of humans present contributing to the encounter rate described above	1000	[26,43]
*P*	Proportion of mosquitoes alive at the start of each hour that survive hazards other than host attack over the course of that hour	0.995	Derived from sensitivity analysis of daily values [26]
*S*_*t*_	Suitability of a given hour-long time interval to the mosquito for host seeking, expressed as a proportion of their maximum host-seeking activity	0 to 1	Assumed to vary over the course of each 24-hour day to reflect one of three distinct stereotypical patterns of preferred feeding times
*t*	An hour-long time interval during the 24-hour day starting at 18:00, corresponding to the intervals 18:00 to 19:00, 19:00 to 20:00 etc., through to 17:00 to 18:00.	0,1,2,3…23	[30]

**Table 2:** Definitions and equations for derived process, state and life history parameters

**Symbol**	**Definition**	**Equation**
*Process parameters*		
*E*_*t*_	Hourly rate at which an unfed host-seeking mosquito encounters a human host during a given hour-long time interval (t) during the 24-hour day	1
*γ*_*t*_	Mean probability of a mosquito attacking an average encountered human host during a given hour-long time interval (t) during the 24-hour day	2
*γ*_*u*_	Mean probability of a mosquito attacking an encountered unprotected human host during a given hour-long time interval (t) during the 24-hour day	3
*γ*_*p*_	Mean probability of a mosquito attacking an encountered human host who is protected by using a long-lasting insecticidal net during a given hour-long time interval (t) during the 24-hour day	4
ϕ_t_	Mean probability of a mosquito attacking and successfully feeding upon an average encountered human host during a given hour-long time interval (t) during the 24-hour day	5
ϕ_u_	Mean probability of a mosquito attacking and successfully feeding upon an encountered unprotected human host during a given hour-long time interval (t) during the 24-hour day	6
ϕ_p_	Mean probability of a mosquito attacking and successfully feeding upon an encountered human host who is protected by using a long-lasting insecticidal net during a given hour-long time interval (t) during the 24-hour day	7
*State parameters*		
*B*_*a*_	Probability that a recruited mosquito bites a single unprotected human during a given age interval *(a)*	12
*D*_*a*_	Probability that a recruited mosquito dies during a given age interval (*a*)	9,11
*F*_*a*_	Probability that a recruited mosquito feeds successfully during a given age interval (*a*)	8,11
*U*_*a*_	Probability that a recruited mosquito remains unfed at the end of a given age interval (*a*)	10,11
*Life history parameters*		
*B*	Mean lifetime measurable bites upon unprotected humans, expressed as total lifetime probability of feeding upon a single unprotected human, or the sum of the probabilities of feeding on a single unprotected person at each age	16
*B*_*t*_	Mean lifetime measurable bites upon unprotected humans, expressed as total lifetime probability of feeding upon a single unprotected human or the sum of the probabilities of feeding on a single unprotected person at each interval of mosquito age *(a)*, that occurs at that time interval (*a* = *24d* + *t*, where t = 0 corresponds to the hour of emergence; 18:00 for mosquitoes emerging at dusk and 24:00 for mosquitoes emerging at midnight).	17
*F*	Mean lifetime feeding success upon all protected and unprotected humans, expressed as the total number of successful feeds per mosquito lifetime or the sum of the probabilities of feeding at each age	13
*F*_*t*_	Mean lifetime feeding success all protected and unprotected at a specific time interval *(t)*, expressed as the total number of successful feeds per mosquito lifetime or the sum of the probabilities of feeding at each interval of mosquito age that occurs at that time interval (*a* = *24d* + *t* where t = 0 corresponds to the hour of emergence; 18:00 for mosquitoes emerging at dusk and 24:00 for mosquitoes emerging at midnight).	14
*π*_*F,i*_	Proportion of successful feeds upon all protected and unprotected humans occurring indoors	15
*π*_*B,i*_	Proportion of measurable bites upon unprotected humans occurring indoors	18

### Simulating nocturnal fluctuations in the behavioural interactions between mosquitoes and humans

Instead of assuming a fixed host encounter rate for increments of entire nights, the rate of encounter of all hosts (E_t_) was distributed across hours of the night (t = 0 ,1,…, 23 denoting the periods 18:00 to 19:00, 19:00 to 20:00, .... and 17:00 to 18:00, respectively [30]) according to assumed, stereotypical *a priori* distributions of suitability (S) of each hour to the mosquito for host-seeking foraging activity, given its innate preferences:

(1) $$E_{t}=E_{\text{max}}S_{t}$$

Where E_max_ represents the maximum hourly host encounter rate under optimal conditions and E_t_ represents the hourly host encounter rate during a specific hour. To estimate E_max_, it was assumed that almost all mosquitoes encountering unprotected hosts attack them (γ → 1, where γ is defined as the probability that a host-seeking mosquito attacks a host after encountering it). Therefore, the total encounter rate per night (E) is approximately represented by the estimate for total host attack availability (E = A/ γ ≈ A where A is defined as the rate at which a single host-seeking mosquito encounters and attacks all hosts) previously reported for Namawalla village under historical conditions of negligible net use [26,31]) and dividing by an assumed mean nightly activity period of eight hours (E_max_ = E/8 ≈ A/8). Thus E_t_ (where t is an integer between 0 and 23) represents the mosquito’s probability of encountering a human at time t.

**Figure 2:**
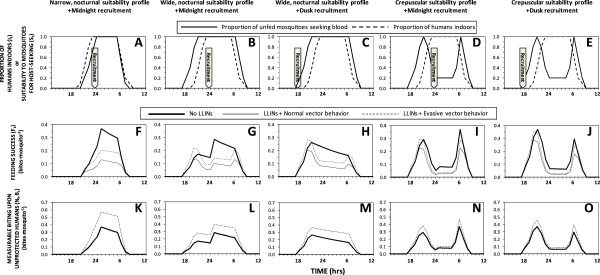
**Lifetime mean distributions of vector feeding success (F to J), and measurable biting activity upon non-users of long-lasting, insecticidal nets (K to O), as a function of preferred feeding suitability profiles and recruitment time (A to E), as well as evasion of insecticide contact once inside houses (dashed versus solid thin lines) under conditions of host availability estimated for *Anopheles arabiensis *in rural Tanzania (Table**1**).** The large arrows denote the times at which all mosquitoes were initially recruited to the adult host-seeking population as described in the methods section.

Three different suitability profiles were assumed: 1) a narrow, strictly nocturnal profile of preferred activity which fits within the peak of preferred hours for humans to sleep indoors (Figure 2A) and may be protected by an LLIN; 2) a wider nocturnal profile that includes two extra hours at dusk and at dawn, during which most humans are awake outdoors and therefore unprotected by LLINs (Figure 2B and C); and, 3) a crepuscular profile with the same early activation and late deactivation trajectories at dusk and dawn but suppressed feeding preference during nocturnal hours when most humans are asleep indoors (Figure 2D and E) and may therefore be protected by an LLIN. In addition, two different recruitment times were assumed for emergence, reflecting two of the most commonly observed stereotypical preferences among a range of mosquito species [32] that have very different implications for mosquito feeding success and survival: 1) emergence at dusk when active mosquitoes may feed freely because humans are also active and unprotected by LLINs (18:00, Figures 2C and E); and, 2) emergence at midnight when feeding opportunities are more restricted because humans are inactive so all users of LLIN are protected by them (24:00, Figures 2A, B and D).

A recent multisite analysis has demonstrated that, while the bulk of human exposure to the major vectors of Africa occurs indoors, this is because peak mosquito-biting activity coincides with times of the night when people are asleep indoors, and the mosquitoes themselves have no substantial or consistent preference for biting indoors or outdoors [2]. It was therefore assumed that the only influence that the proportion of humans indoors has on host-seeking or feeding events is the possibility that the human may use a net. The proportion of time spent indoors but not sleeping is typically very small in African communities [2,33] and is assumed to be negligible in the interests of simplicity. Being indoors is therefore assumed to be equivalent to using and being protected by a net for the proportion of the population that report using an LLIN. To enable further simplification, the mosquito population in question was also assumed to utilize only human hosts, as is usually the case for the most potent African primary vectors, such as *An. funestus* and *An. gambiae *[34].

Additionally, human behaviour also varies predictably across diurnal cycles. The proportion of humans who are asleep indoors (I_t_) at given time (t, defined as described above for E_t_), where they can conveniently use a net, is typically high during hours of darkness when most people sleep (I_t_ → 1) and low during daylight hours when most people are active (I_t_ → 0). Similar to E_t_, I_t_ represents the probability that a human is indoors and, if using a net, protected at a given time (t). The probability that a mosquito attacks an encountered host at a given time (γ_t_) is defined as the complement of the probability of diverting away from it, and this probability can also be calculated as the mean of the attack probabilities for protected and unprotected hosts, weighted by the proportions of humans using (C_h_) or not using (1-C_h_) them:

(2) $$\begin{array}{l} \gamma_{t}=1-\Delta_{t}=C_{h}I_{t}\gamma_{p}+\left(1-C_{h}I_{t}\right)\gamma_{u}\\ \;=C_{h}I_{t}\left(1-\Delta_{p}\right)+\left(1-C_{h}I_{t}\right)\left(1-\Delta_{u}\right) \end{array}$$

Where the probabilities of attacking a protected (γ_p_) and unprotected (γ_u_) human host are defined as follows:

(3) $$\gamma_{u}=\left(1-\Delta_{u}\right)$$

(4) $$\gamma_{p}=\left(1-\Delta_{p}\right)$$

And the probabilities of a mosquito diverting away from attacking a human host that is either protected by using an LLIN (Δ_p_) or unprotected (Δ_u_) at that time are defined exactly as previously described [26].

The probability that a mosquito attacks an encountered host and successfully feeds on it at a given time (*ϕ*_t_) is similarly calculated as the mean of the feeding probabilities for protected and unprotected hosts, weighted by the proportions of humans using or not using them:

(5) $$\phi_{t}=C_{h}I_{t}\phi_{p}+\left(1-C_{h}I_{t}\right)\phi_{u}$$

Where the probabilities of feeding upon a protected (*ϕ*_p_) and unprotected (*ϕ*_u_) human host are respectively defined as the products of the probabilities of attacking and surviving that attack:

(6) $$\phi_{u}=\gamma_{u}\left(1-\mu_{u}\right)=\left(1-\Delta_{u}\right)\left(1-\mu_{u}\right)$$

(7) $$\phi_{p}=\gamma_{p}\left(1-\mu_{p}\right)=\left(1-\Delta_{p}\right)\left(1-\mu_{p}\right)$$

And the probabilities that a mosquito dies as a result attacking humans that are either protected by using an LLIN (μ_p_) or unprotected (μ_u_) at that time are defined exactly as previously described [26].

Given these assumptions and background parameters, the fate of mosquitoes of any given age expressed in hours (a) can be described by breaking down the proportion of all recruited mosquitoes that survived unfed to the previous age, in terms of the derived fractions of the original recruited cohort that survived unfed, survived but fed successfully, or died during the following hour (Figure 1). The proportion of all recruited mosquitoes which survived and successfully fed since the preceding age (F_a_) is defined as the product of the proportion of unfed mosquitoes of the preceding age (U_a-1_), the mean hourly probability of surviving all mortality hazards other than host attack (P), the encounter rate of human hosts over that time period (E_t_), and the proportion that are neither diverted nor killed and therefore successfully feed (*ϕ*_t_) following encounter of a human:

(8) $$F_{a}=U_{a-1}P\;E_{t}\phi_{t}$$

With *t ≡ a* modulo 24 for mosquitoes assumed to be recruited at dusk and *t ≡ (a + 18)* modulo 24 for mosquitoes assumed to be recruited at midnight. Since *a* represents the age of the mosquito in hours (with a value between 0 and 720) and *t* represents the time of day (as described above with a value between 0 and 23), the modulo operator converts the age of the mosquito into the time of day. Since time *t = 0* represents 18:00, for mosquitoes emerging at 18:00, the conversion of the age is simply the remainder after dividing *a* by 24. For mosquitoes that emerge at midnight, an addition of 18 is needed to convert the age to the time of day.

The proportion of all recruited mosquitoes that died during the preceding hour is calculated as the product of the proportion of unfed mosquitoes of the preceding age (U_a-1_) and the sum of mortality probabilities arising from attacking protected (μ_p_) and unprotected (μ_u_) hosts and other hazards (1-P), with the former being conditional upon surviving those other hazards (P), encountering a human host (E_t_), and attacking them (γ_p_ and γ_u_, respectively):

(9) $$D_{a}=U_{a-1}\left(\left(1-P\right)+P\;E_{t}\left(C_{h}I_{t}\gamma_{p}\mu_{p}+\left(1-C_{h}I_{t}\right)\gamma_{u}\mu_{u}\right)\right)$$

Again with *t* ≡ *a* modulo 24 for mosquitoes assumed to be recruited at dusk and *t ≡ (a + 18)* modulo 24 for mosquitoes assumed to be recruited at midnight.

For mosquitoes that are too young to have completed a full oviposition cycle (two days old or less), the proportion of all recruited mosquitoes that remain unfed is calculated as the product of the proportion of unfed mosquitoes of the preceding age *(U*_*a-1*_*)*, the mean hourly probability of surviving all mortality hazards other than host attack *(P)*, and the sum of the probabilities that a mosquitoes will not encounter a human host *(1-E*_*t*_*)* or encounter but divert away from attacking one *(E*_*t*_*Δ*_*t*_*)*:

(10a) $$U_{a}=U_{a-1}P\;\left(\left(1-E_{t}\right)+E_{t}\;\Delta_{t}\right),\;\text{where}\;a\leq 48$$

And for older cohorts, the proportion of all recruited mosquitoes that have survived and are unfed must also account for the return to an unfed state of mosquitoes that successfully fed two days previously and survived the assumed intervening gestation period of 48 hours:

(10b) $$\begin{array}{l} U_{a}=U_{a-1}P\;\left(\left(1-E_{t}\right)+E_{t}\;\Delta_{t}\right)+F_{a-48}P^{48}\\ \;\text{where}\;a>48 \end{array}$$

Again, with *t* ≡ *a* modulo 24 for mosquitoes assumed to be recruited at dusk and *t ≡ (a + 18)* modulo 24 for mosquitoes assumed to be recruited at midnight.

Note that the sum of the proportions of mosquitoes which fed, died or remained unfed over the duration of one hourly age interval is equal to the proportion remaining unfed at the end of the previous age interval up to the age of two days:

(11a) $$U_{a}+F_{a}+D_{a}=U_{a-1}\;\text{for}\;a\leq 48$$

And that for older mosquitoes an additional contribution is made by those that fed 48 hours previously, survived and re-enter the unfed state at this age, immediately after laying their eggs:

(11b) $$U_{a}+F_{a}+D_{a}=U_{a-1}+F_{a-48}P^{48},\text{for}\;a>48$$

With initial conditions defined as U_0_ = 1 and F_0_ = D_0_ = 0, since all mosquitoes are assumed to emerge unfed.

In order to plot a longitudinal picture of measurable biting rates upon human volunteers in human landing catches, the probability that an unprotected human lacking a net would be bitten by a recruited mosquito is also calculated for each age (B_a_) based on hourly encounter rates and feeding probabilities at a given time of the night, with the former divided by the size of the human population (N_h_):

(12a) $$B_{a}=U_{a-1}P\;E_{t}\phi_{u}/N_{h}$$

For the purposes of presentation with larger proportions and less decimal places, this parameter is also expressed in relation to the probability of biting anyone from the entire human community if any protection they have is removed at the start of that age:

(12b) $$B_{a}N_{h}=U_{a-1}P\;E_{t}\phi_{u}$$

### Mosquito life history summary parameters

In addition to the hour-by-hour predictions of equations (1) to (12b), a number of important life table summary outcomes are also calculated, assuming that the contributions of mosquitoes more than 30 days or 720 hours old are negligible [35]. The mean lifetime feeding success (F) is calculated as the total number of successful feeds per mosquito lifetime or the sum of the probabilities of feeding at each age:

(13) $$F=\sum_{a=1}^{720}F_{a}$$

Breaking this lifetime success rate down to rates for specific times of that night (t) that the feeding occurs (F_t_) requires breaking the age of the mosquito in hours (a) into the age of the mosquito expressed as an integer number of days (d) plus the remaining hours (a = 24d + t):

(14a) $$F_{t}=\sum_{d=1}^{30}F_{\left(a=\right)24d},\;\mathrm{for}\;t=0$$

(14b) $$F_{t}=\sum_{d=0}^{29}F_{\left(a=\right)24d+t},\;\mathrm{for}\;1\leq t\leq 23$$

In equations (14a and 14b), *t = 0* corresponds to the hour of emergence (18:00 for mosquitoes emerging at dusk and 24:00 for mosquitoes emerging at midnight).

The proportion of successful feeds occurring indoors is calculated based on the previously described parameters, as well as the specific feeding probabilities for humans while protected (*ϕ*_p_) and unprotected (*ϕ*_u_) by an LLIN:

(15) $$\pi_{F,i}=\frac{\sum_{a=1}^{a=720}\left[U_{a}E_{t}I_{t}\left(C_{h}\phi_{p}+\left(1-C_{h}\right)\phi_{u}\right)\right]}{\sum_{a=1\;}^{a=720}\left[U_{a}E_{t}\left(I_{t}\left(C_{h}\phi_{p}+\left(1-C_{h}\right)\phi_{u}\right)+\left(1-I_{t}\right)\phi_{u}\right)\right]}$$

Equivalent terms for overall (B) and time-specific (B_t_) total probabilities of biting a unprotected human per mosquito lifetime are calculated as follows:

(16) $$B=\sum_{a=1}^{720}B_{a}$$

And:

(17a) $$B_{t}=\sum_{d=1}^{30}B_{\left(a=\right)24d}\;\mathrm{for}\;t=0$$

(17b) $$B_{t}=\sum_{d=0}^{29}B_{\left(a=\right)24d+t}\;\mathrm{for}\;1\leq t\leq 23$$

Again, similar to equations (14), in equations (17), *t* = *0* corresponds to the hour of emergence (18:00 for mosquitoes emerging at dusk and 24:00 for mosquitoes emerging at midnight).

The average proportion of the bites upon unprotected humans that occurs indoors (π_B,i_ which was previously denoted π_i_[2,30] but should not be confused with the superscripted crude binomial estimate for this parameter, π^B^_i_[2]), was calculated by weighting the mean probabilities of survival and attack for each hour of the night (t) by the proportion of humans reporting to have been indoors (I_t_) and outdoors (1- I_t_), respectively, at that time:

(18) $$\pi_{B,i}=\frac{\sum_{a=1}^{a=720}\left[U_{a}E_{t}I_{t}\phi_{u}\right]}{\sum_{a=1\;}^{a=720}\left[U_{a}E_{t}\phi_{u}\right]}$$

The terms B, B_a_ and B_t_ all reflect total or subtotal probabilities that an unprotected human resident would be bitten by an average mosquito over its lifetime so they are directly proportional to field measurements of biting rates on unprotected volunteers using the established human landing catch technique [36,37]. Correspondingly, the calculated proportion of all mosquito bites upon unprotected humans occurring indoors (π_B,i_) is also equivalent to field estimates of this epidemiologically important quantity based on human landing catch measurements [2].

Note also that similar summary variables were calculated for the older age groups (>ten days) of mosquitoes that mediate malaria transmission but these outcomes were essentially identical to those calculated for the entire life span so these results are not presented.

### Parameterization

The values used for all base input parameters are summarized in Table 1. Assumed values for total host availability, and therefore encounter rate (E ≈ A = 1.45 attacks or encounters per host-seeking mosquito per night, equivalent to 0.181 attacks or encounters · host-seeking mosquito^-1^ · hour^-1^ for an eight hour night), human population size (N_h_ = 1000), hourly probability of surviving hazards other than host attack (P = 0.995), and LLIN coverage (C_h_ = 0.8), as well as the encounter-associated diversion probability (Δ_u_ = 0.1) and attack-associated mortality probability (μ_u_ = 0.1) for unprotected humans, were chosen to maintain consistency with previous simulations and the assessments of their probable values [26,31,38]. The encounter-associated diversion probability (Δ_p_) and the attack-associated mortality probability (μ_p_) for protected humans were both assumed to be reasonably high for typical vectors exhibiting behaviors within houses that are similar to *An. gambiae* and *An. funestus* (Δ_p_ = μ_p_ = 0.6) [14]. However, for a vector exhibiting the kind of intradomiciliary evasive behaviour that has recently been reported for *An. arabiensis *[14-16], greater diversion (Δ_p_ = 0.8) and lower mortality (μ_p_ = 0.2), resulting in the same level of overall personal protection (ρ = 1-[((1- Δ_p_)(1- μ_p_))/((1- Δ_u_)(1- μ_u_))] = 0.802), was assumed. In the interests of simplicity, all attack-associated mortality was assumed to occur before feeding so that distinct pre-feed and post-feed mortality terms [26] were not required.

## Results

### Effects of insecticidal nets upon temporal distributions of feeding success for mosquitoes with distinct behavioural preference profiles

**Figure 3:**
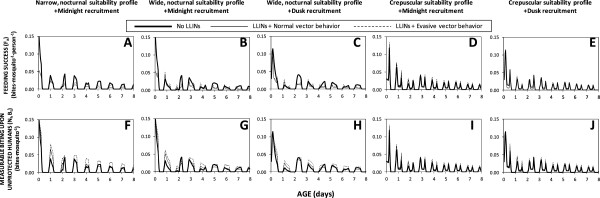
**Distributions of vector feeding success (A to E), and measurable biting activity on non-users of long-lasting insecticidal nets (F to J), over the first eight days of life (30 days were simulated but are not presented in the interests of clarity) as a function of preferred feeding suitability profiles, recruitment time and evasion of insecticide contact once inside houses, under conditions of host availability estimated for *Anopheles arabiensis *** **in rural Tanzania (Table**1**).**

Figure 2 illustrates the variety of effects that LLINs can have upon actual feeding success and measurable biting activity of mosquito populations with a range of pre-existing behavioural traits. For a vector population with a narrow, nocturnal window of preferred activity, during which vast majority of humans are asleep, LLINs are predicted to reduce feeding success by 69% where they achieve reasonable levels of mosquito mortality per attack, but only by 38% if the mosquito evades fatal contact with the treated surface of the net (Figure 2F). Little impact upon the distribution of feeding success across the night is predicted because the mosquito is trapped within its preferred feeding activity period so feeding success by surviving unfed mosquitoes is redistributed to similar times on subsequent nights, especially if the vector evades insecticide contact (Figure 3A). For a vector with a preferred feeding activity window that also peaks in the middle of the night, but which extends sub-optimally for an hour after and two hours before humans go indoors to sleep (Figures 2B and C), feeding success is clearly redistributed by LLIN use during sleeping hours to defined peaks at dawn and dusk when LLIN use is low, especially where the vector evades fatal contact with the treated net surface (Figures 2G, H and 3B, C). In an otherwise identical scenario where recruitment to the adult host-seeking population occurs in the early evening rather than at midnight, a greater peak of feeding success occurs at dusk than at dawn because mosquitoes begin their first host-seeking phase before humans go to sleep and begin using LLINs (Figures 2H and 3C). For a crepuscular mosquito which down-regulates host-seeking activity when most humans are asleep, remaining peaks of foraging suitability coincide with availability of unprotected humans outdoors so little redistribution of feeding success occurs. In such a scenario, LLINs reduce feeding success by less than half for mosquitoes that do not evade fatal insecticide contact after entering houses and less than a quarter for those that do, regardless of whether recruitment occurs at dusk or midnight (Figures 2I, J and 3D, E).

### Effects of preferred feeding time patterns upon lifetime feeding success in the presence of insecticidal nets

**Figure 4:**
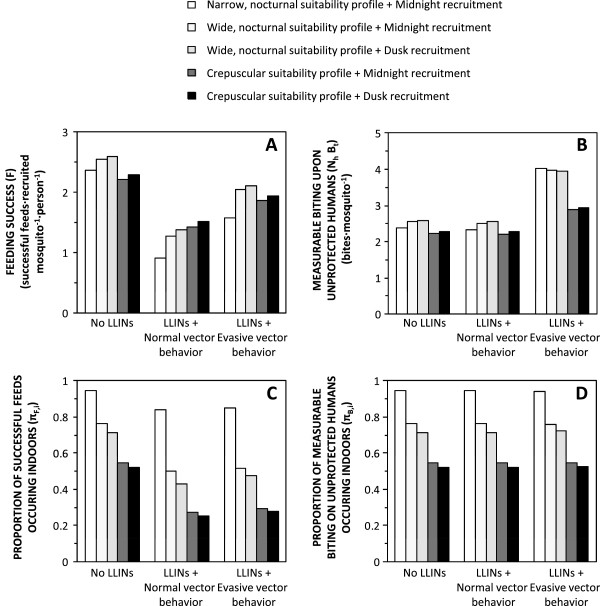
**Vector lifetime total feeding success (A), and measurable biting activity upon non-users of long-lasting insecticidal nets (B), as well the proportion of these totals which occur indoors (C and D, respectively), as a function of preferred feeding suitability profiles, recruitment time and evasion of insecticide contact once inside houses, under conditions of host availability estimated for *Anopheles arabiensis *** **in rural Tanzania (Table **1**).**

The predicted advantages and disadvantages to the mosquito of alternative innately preferred feeding and recruitment times appear marginal in the absence of LLINs (Figures 4A and C). However, the predicted impact of these heritable preferences upon feeding success in the presence of LLINs clearly illustrates the importance of behavioural resilience or resistance traits that enable vectors to avoid or survive otherwise fatal encounters with them (Figures 4A and C). Overall, LLINs consistently reduce overall feeding success (Figure 4A) and the proportion of successful feeds that occur indoors (Figure 4C). However, LLIN impact is attenuated by all the simulated phenotypic preferences for times of behavioural activity that enable the mosquito to feed at times when humans are outdoors and nets are not used: recruitment to the adult population at dusk, extension of nocturnal activity to dawn and dusk and reduced activity during sleeping hours when humans are indoors (Figure 4A). However, it is notable that the ability to avoid fatal contact with treated surfaces after entering houses, as recently reported for *An. arabiensis* in Tanzania [14-16] and decades ago for a range of American vectors [22], is consistently predicted to be a more important resilience or resistance trait than any of these preferences for feeding and recruitment times (Figure 4A).

### Effects of preferred feeding time profile upon the distribution of biting activity on unprotected humans

Outside of specially designed experimental huts, it is not practical to directly measure *de facto* feeding success rates of mosquitoes with current entomological methodologies [37], so evaluations of their feeding activity distributions usually relies on landing catches conducted by unprotected human subjects. While these field surveys are not entirely representative of mean human exposure because they are conducted by adult males who stay seated and awake all night, the biting rates observed with this technique should be consistently proportional to the rates at which host-seeking mosquitoes attack humans and succeed in biting non-users of LLINs, rather than the average for the human population as a whole. Contrary to the hypothesis that motivated this modelling analysis [3], it is notable that predictions of such attack rate distributions indicate little change in expected patterns of observable host-seeking activity (Figures 2K to O). In the presence of LLINs, the distribution of measurable biting activity is clearly shifted to later nights (Figures 3F to J) without any obvious change in the distribution across times of the night (Figures 2K to O and 3F to J). Reductions in survival as mosquitoes age are counterbalanced by an increase in the proportion of surviving mosquitoes that remain unfed and attack unprotected hosts, especially while other residents are safe under their LLINs, so measurable biting rates in the presence of nets often spike above those in the absence of LLINs, especially for vectors with evasive behaviour (Figures 3F to J). For vectors exhibiting normal behaviours, reduced survival and increased proportions of biting upon the minority of unprotected humans balance each other out almost exactly: the biting profiles that would be observed by a human landing catch participant in the presence of LLINs are so numerically similar (but not identical) to those in the absence of LLINs that they are essentially indistinguishable in Figures 2K to O and 4B to D. Furthermore, overall increases of the number of bites experienced by unprotected humans are predicted for vector populations which evade fatal insecticide contact and therefore make more numerous, less successful but also less hazardous, attacks per night and per feeding cycle (Figures 3F to J), as well as per lifetime (Figures 2K to O and 4B and D). Even though overall feeding success of mosquitoes is consistently reduced by LLINs (Figures 2F to J and 4A), measured biting rates may be unaffected or even increased (Figures 2K to O and 4B) because human landing catch participants and other unprotected non-users of LLINs account for a disproportionate share of feeding opportunities available to mosquitoes prevented from feeding by LLINs, especially during hours when most residents are asleep and protected. So although LLIN coverage does increase the proportion of mosquitoes that remain alive, unfed and active at dawn and dusk, biting rates experienced by unprotected humans during sleeping hours are also increased because community-wide LLIN use prevents mosquitoes from feeding and leaving the host-seeking state. The overall result is therefore a redistribution of accumulated activity across the preferred temporal activity profile of the mosquito so that no substantive change in attack rate profile is predicted (Figures 2K to O, 3F to J, 4B and D).

Note that when analysis was restricted to events occurring after mosquitoes pass the age of ten days, the predictions obtained were essentially identical to those depicted in Figures 2 and 3 so these results are not presented. It therefore seems unlikely that alterations of measurable attack distributions could occur among older, infected mosquitoes unless their behaviour is directly influenced by associated factors such as learning [44-46] or infection status [47-49], rather than just the feeding life history events captured by this model.

### Sensitivity of conclusions to variations in the availability of human hosts

**Figure 5:**
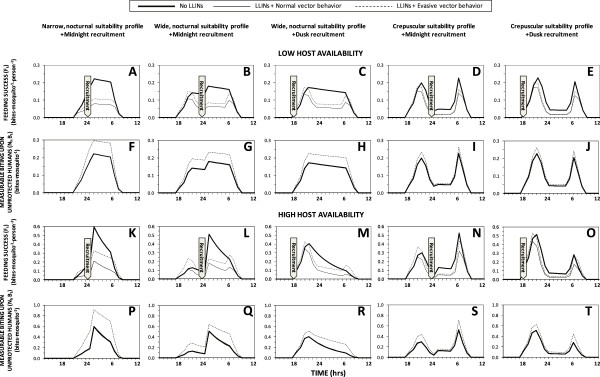
**Lifetime mean distributions of vector feeding success (A to E and K to O), and measurable biting activity upon non-users of long-lasting insecticidal nets (F to J and P to T), as a function of preferred feeding suitability profiles, recruitment time and evasion of insecticide contact once inside houses, under conditions of lower (×1/3) and higher (×3) host availability, and therefore maximum encounter rates (E**_**max**_**), than field estimates for *Anopheles arabiensis *in rural Tanzania (Table **1**).** The large arrows denote the times at which all mosquitoes were initially recruited to the adult host-seeking population as described in the methods section.

**Figure 6:**
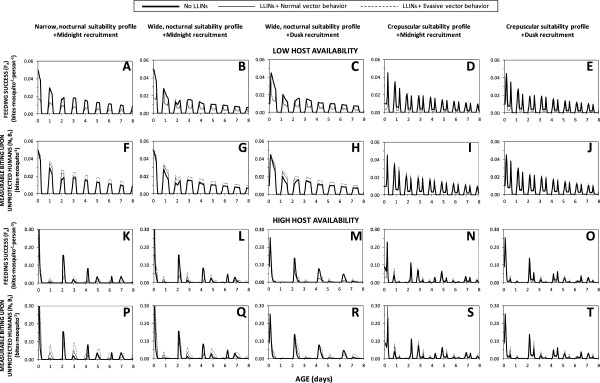
**Distributions of vector feeding success (A to E and K to O), and measurable biting activity (F to J and P to T) on non-users of long-lasting insecticidal nets, over the first eight days of life (30 days were simulated but are not presented in the interests of clarity) as a function of preferred feeding suitability profiles, recruitment time and evasion of insecticide contact once inside houses, under conditions of lower (×1/3) and higher (×3) host availability, and therefore maximum encounter rates (E**_**max**_**), than field estimates for *Anopheles arabiensis *** **in rural Tanzania (Table **1**).**

**Figure 7:**
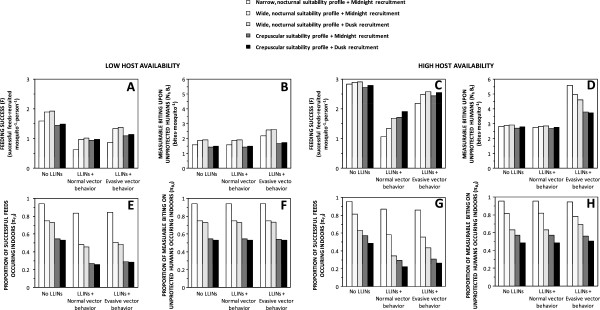
**Vector lifetime total feeding success (A and C), and measurable biting activity upon non-users of long-lasting insecticide-treated bed nets (B and D), as well the proportion of these totals which occur indoors (E, F, G and H), as a function of preferred feeding suitability profiles, recruitment time and evasion of insecticide contact once inside houses under conditions of lower (×1/3) and higher (×3) host availability and therefore maximum encounter rates (E**_**max**_**), than field estimates for *Anopheles arabiensis *** **in rural Tanzania (Table **1**).**

The way that patterns of host-seeking activity and feeding success by mosquito populations change following LLIN introduction might also depend on how rapidly they can find humans, especially at times when most people are outdoors, awake and do not use nets. The sensitivity of the model predictions to variations in human host density was therefore examined by repeating these simulations with three-fold lower or higher values for total host availability. Lowering host availability consistently strengthens the synchronizing influence of mosquito feeding time preference upon the distribution of host-seeking activity, regardless of LLIN use (Figures 5, 6 and 7). Lower host availability also has little influence upon the redistribution of feeding success because mosquitoes find it even more difficult to utilize the opportunities presented by non-users of LLINs (Figures 5, 6 and 7). At higher host availability, feeding activity becomes more clearly synchronized by recruitment time, even in the presence of LLINs, because feeding occurs more rapidly after recruitment (Figures 5, 6 and 7). Greater redistribution of feeding success to non-users and waking hours when net use is low, as well as reduced overall impact on feeding success, was only consistently predicted for vector populations capable of evading fatal contact with nets (Figures 5, 6 and 7). Otherwise, these increased encounter rates led to increased mortality rates at times when nets are in use so that fewer survive until times when humans are largely outdoors and unprotected (Figure 6). While LLIN scale up in settings with high host availability has some influence upon the proportion of exposure of non-users of LLINs that occurs indoors, these changes are neither consistent nor of substantive magnitude (Figures 5, 6 and 7).

## Discussion

Overall, these simulations suggest that recent reports of dramatically altered malaria vector biting activity patterns, measured as landing rates on unprotected human participants rather than rates of actual community-wide feeding success, cannot be explained in terms of simply extending foraging activity until humans are awake and fully exposed at dawn and dusk. However, field observations of altered feeding activity profiles may be parsimoniously rationalized in terms of a heritable extension of strictly nocturnal preferred feeding times, to also include dawn and dusk, or even crepuscular patterns wherein host-seeking activity is limited to dawn and dusk. It therefore appears plausible that genuine behavioural resistance traits are now emerging in residual vector populations that have historically expressed sufficient behavioural resilience traits to persist and evolve in the face of high coverage with LLINs or IRS.

On the other hand, recent convincing experimental evidence of associative learning in mosquitoes [44-46] suggests an alternative or additional explanation: over the course of their lives, mosquitoes may learn where and when humans are exposed under conducive conditions for feeding upon them, and time their host-seeking activities accordingly. Although this model does not attempt to capture such complex evolutionary or learning processes, the emergence of such behavioural resistance traits or phenotypic plasticity of preferred feeding times may be conceptualized in terms of evolutionary selection or associative learning of the survival-optimizing behavioural preferences illustrated in the right hand panels of Figures 2 and 3 and the right hand bars of Figure 4, to replace behavioural preferences to the left that render mosquitoes more vulnerable to LLINs and IRS. While more advanced modelling studies to test the plausibility of such explanatory hypotheses are desirable and should be encouraged, definitive evaluation of the underlying causes of shifting biting activity patterns will ultimately require lucid experiments and field observations with real mosquitoes.

Regardless of whether such behaviours are classified as phenotypically plastic expression of pre-existing resilience traits, or as evolutionary selection of heritable resistance traits, they need to be meaningfully addressed if elimination of malaria from the tropics is ever to become a reality [3,5,23]. The ability to avoid entering houses by seeking hosts at dawn and dusk, or even down-regulating host-seeking activity at night when people are indoors, can clearly limit the impact of LLINs so this gap in biological coverage [50] must be filled with complementary interventions such as spatial repellents or insecticide-treated clothes [3,5,6,13,23,25,27,51-54].

However, perhaps the most important conclusion suggested by Figures 2 to 4 is that evasion of fatal insecticide contact following house entry, presumably by minimizing exposure to treated surfaces while feeding or resting [21,22] and exiting soon after entering [15,22], may be a more important form of resilience or resistance than preferences for emerging or feeding at dawn and dusk when humans are unprotected. This is particularly worrying because such behavioural phenotypes defeated historical attempts to eliminate malaria transmission by several predominantly indoor-feeding Latin American vectors [22]. The ability to safely enter and exit houses containing IRS or LLINs, without incurring fatal exposure to insecticide-treated surfaces, has now been documented twice in *An. arabiensis,* one of the most important vectors of residual malaria transmission in Africa [14-16]. It is therefore understandable this species has maintained its historically observed nocturnal peak of biting activity [55,56] so that most exposure of residents lacking LLINs, and as much as half for LLIN users [25], continues to occur indoors at two separate locations in western Kenya [2,17] and southern Tanzania [2,8,57] where high coverage of insecticidal nets has been in place for several years. It is also striking that Elliott noted how all the most potent vectors of malaria in the Americas consistently and persistently exhibited nocturnal biting patterns coinciding with times of the night when most people are asleep indoor, even following the introduction of IRS, while less dangerous *Anopheles* species all exhibited crepuscular biting patterns [22], presumably reflecting a lack of specialist adaptation to feeding upon humans when they are asleep.

Of particular concern is the potential for behavioural phenotypes which minimize insecticide contact duration to synergize with complementary forms of physiological resistance, such as the modified integument phenotypes that presumably slow penetration of insecticides to target tissues in *An. funestus*, *An. gambiae* and *Anopheles stephensi *[24,58], as well as bed bugs [59]. Such intradomiciliary evasiveness may also represent a form of behavioural resilience or resistance with lower fitness costs than the altered feeding time preferences, which were conspicuous by their apparent absence from recently assessed populations of *An. arabiensis* in Kenya and *An. funestus* in Zambia in settings with high LLIN coverage [2,60]. Suppression of host-seeking activity during sleeping hours obviously limits the amount of time available for mosquitoes to obtain blood, while extension of feeding preferences to include dawn and dusk may incur higher mortality rates by forcing mosquitoes to operate outside of their historical comfort zones of temperature and humidity. While it is likely that mosquito populations across the tropics will exhibit diverse combinations of behavioural and physiological resistance to cope with IRS and LLINs [12], evasion of treated surfaces inside houses by persistently endophagic vectors merits particular attention.

Fortunately, persistent endophagy also presents valuable opportunities to tackle such important primary vectors with improved technologies for indoor vector control [60]. While the elimination of malaria from historically holo-endemic regions of the tropics will undoubtedly necessitate the extension of vector control beyond houses [1,3-6,8,11-13,23,25,27,50-54], eliminating residual indoor transmission is probably equally important [60]. In fact, residual transmission in areas with high LLIN coverage is approximately equally distributed indoors and outdoors [25]. Crucially, the indoor environment presents unique opportunities for vector control because solid-phase insecticides which kill mosquitoes on contact, but obviously require surfaces to apply them to, are likely to have greater impact for a given level of coverage and protective efficacy than vapour-phase spatial repellents which can be applied in absence of a surrounding structure but merely divert mosquitoes elsewhere [27]. Structural surfaces within human habitations also allow application of a wider variety of pesticides than can be safely delivered to human skin or clothing, or to the air we breathe [27]. Unfortunately, varying the active ingredient class used for IRS appears to have little impact on *An. arabiensis* because its resilience or resistance to control by this approach is predominantly behavioural rather physiological [14-16]. More encouragingly, examples of efficacious prototype products for killing high proportions of *An. arabiensis*, in settings where they are known to exhibit intradomiciliary evasive traits, have been described [61,62].

Netting baffles, placed around the eave gaps between the roof and the top of the wall, are commonly used in experimental huts to allow mosquitoes to enter but then prevent their exit [37,63,64]. Eave baffles slant upwards and inwards from the top of wall of the house towards the roof but leave a small gap, so that mosquitoes entering via the eaves are not deterred but gently funnelled towards this narrower entrance that is more difficult for them to find when exiting [37,63,64]. These have recently been evaluated as a control measure in their own right by treating them with carefully optimized, non-repellent formulations of entomopathogenic fungi [65,66] which do not require long contact times because they achieve fatal doses through post-exposure growth rather than absorption by diffusion [61]. By combining this biological insecticide with a minor structural housing modification that turns houses into traps, it has been possible to force even this, otherwise robust, species into fatal contact with treated surfaces [61]. Furthermore, another prototype device that was tested in the same setting demonstrates how the efficacy of existing “off-the-shelf” formulations of conventional chemical insecticides can be dramatically enhanced by simply retaining mosquitoes for long enough within a treated structure [62]. The Ifakara Odour-Baited Station is a simple box baited with synthetic human odours rather than live humans, with several cloth-baffled openings so that mosquitoes can enter but are then impeded from exiting freely [62]. When the organophosphate pirimiphos methyl was applied to cloth or netting panels hung inside this device, far higher proportions of *An. arabiensis* were killed [62] than when the same commercial formulations were applied in a conventional IRS format to the walls and ceilings of experimental huts with unbaffled exit traps into which mosquitoes could rapidly escape [15,16].

These two prototypes are merely examples of how persistently endophagic, behaviourally resilient or resistant vectors may be more effectively tackled by interfering with their ability to exit from host-baited structures, before fatal exposure to insecticides applied within them can occur. Despite their limitations, these examples do illustrate how it may well be possible to substantially improve upon the levels of indoor transmission control that are currently possible with IRS or LLINs [60]. By optimizing and evaluating existing insecticides and delivery formats, to maximize the mortality of mosquitoes entering human dwellings, it should be possible to dramatically enhance control of malaria vectors that feed or rest indoors, within years rather than decades.

## Competing interests

The authors declare that they have no competing interests. The funders had no role in study design, analysis, decision to publish, or preparation of the manuscript.

## Authors’ contributions

GFK conceived the study, formulated the first draft model, executed the simulations and drafted the manuscript. NC reviewed, revised and refined the model and manuscript draft. Both authors read and approved the final manuscript.
